# Epithelioid Rhabdomyosarcoma at the High Parietal Area of the Head: A Case Report

**DOI:** 10.3390/medicina58070951

**Published:** 2022-07-19

**Authors:** Susanne Jung, Lauren Bohner, Miriam Schulte, Johannes Kleinheinz

**Affiliations:** 1 Department of Cranio and Maxillofacial Surgery, University Hospital Münster, 48149 Münster, Germany; susanne.jung@ukmuenster.de (S.J.); johannes.kleinheinz@ukmuenster.de (J.K.); 2 Gerhard-Domagk-Institute of Pathology, Faculty of Medicine, University Hospital Münster, 48149 Münster, Germany; miriam.schulte@ukmuenster.de

**Keywords:** rhabdomyosarcoma, pathology, tumor

## Abstract

Epithelioid rhabdomyosarcoma is a rare condition, which may be clinically misinterpreted as melanoma due to its morphological appearance. Careful morphological and immunohistochemical analysis play an important role in its diagnosis. This case report describes the clinicopathological features of an epithelioid RMS diagnosed at the high parietal area of the head. A 71-year-old male patient presented a red-brown pigmented ulcerative nodule in the high parietal region of the head. Previous biopsy and computer tomography imaging revealed a malignant melanoma in stage I (pT2, sN0, Mx). After tumor operation, histological and immunohistochemical analysis of the tumor were conducted. Histological analysis showed an erosive lesion with a monomorphic cell population containing small cells with prominent nucleoli. A positivity was confirmed for CD10, Vimentin, and Desmin. MyoD1 was detected, as well as a fluctuating signal for p53. Molecular analysis revealed a negativity for Sox-10, and a weak positivity for CK8/18 by absence of p40. Based on the morphological and immunohistochemical findings, the tumor was diagnosed as epithelioid RMS.

## 1. Introduction

Rhabdomyosarcoma (RMS) is a soft tissue malignancy tumor that commonly affects children and adolescents. Currently, four subgroups are known: embryonal, alveolar, pleomorphic, and sclerosing [1,2,3]. Each subgroup differs in their clinicopathological and biological features, and the five-year survival rate ranges between 65–82% [2].

Recently, a new RMS entity, known as epithelioid rhabdomyosarcoma, has been described in the literature [2]. Unlike other subgroups, this variant has been predominantly seen in adults between 60–70 years. The tumor is considered aggressive and it may involve dermis, both dermis and subcutis, or solely the subcutis [4]. 

Epithelioid RMS is a rare condition, which may be clinically misinterpreted as a melanoma due to its morphological appearance [2]. Thus, molecular analysis plays an important role in detecting skillet muscle differentiation [5]. As differential diagnoses, aggressive neoplasms and poorly differentiated carcinoma should be considered [3,4]. 

This paper aims to describe the clinicopathological features of an epithelioid RMS diagnosed at the high parietal area of the head. This case report was conducted according to CARE guidelines [6].

## 2. Case Report

A 71-year-old male patient was referred to the Department of Oral and Maxillofacial Surgery, University Hospital Münster, with a nodule at the high parietal region of the head. Anamnesis revealed trigeminus neuralgia and multiple sclerosis as underlying conditions. No history of allergic reactions or cases of neoplasia within the family were ascertained. Clinically, the patient presented in good health condition. 

Clinical examination revealed a red-brown pigmented ulcerative nodule measuring approximately 1 cm. A biopsy and computer tomography imaging were already conducted alio loco and revealed as diagnosis a malignant melanoma in stage I (pT2, sN0, Mx). 

The surgical procedure was conducted under general anesthesia. An incision was made at the retroauricular area. The tumor was dissected with a marginal safe distance of 1 cm until achieving subcutaneous tissues. A lymph nodes biopsy was performed applying lymph scintigraphy. The soft tissue defect was covered with Syspur-Derm (Leichtform) and the wound was closed using absorbable suture material (Vicryl 2.0). Surgical specimen was assessed by means of histological and immunohistochemical analysis. Pathological findings and diagnosis, such as differential diagnosis, were discussed during a conciliary meeting with pathologists.

## 3. Results

**Figure 1:**
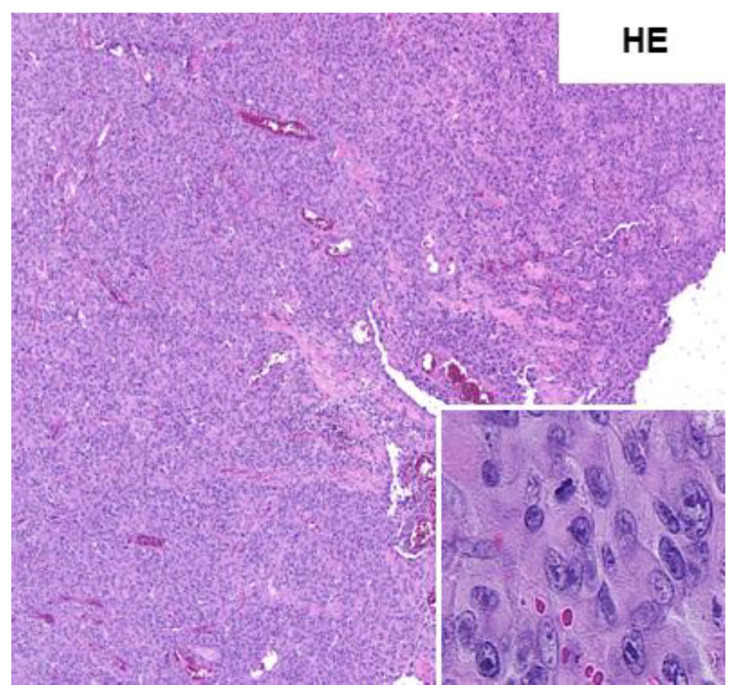
Histological analysis showed cells with prominent nucleoli.

**Figure 2:**
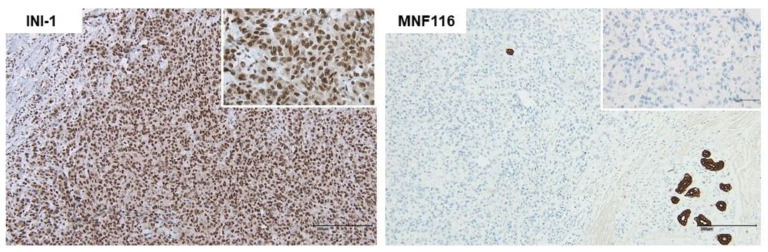
Immunhistochemical staining of cytokeratins.

**Figure 3:**
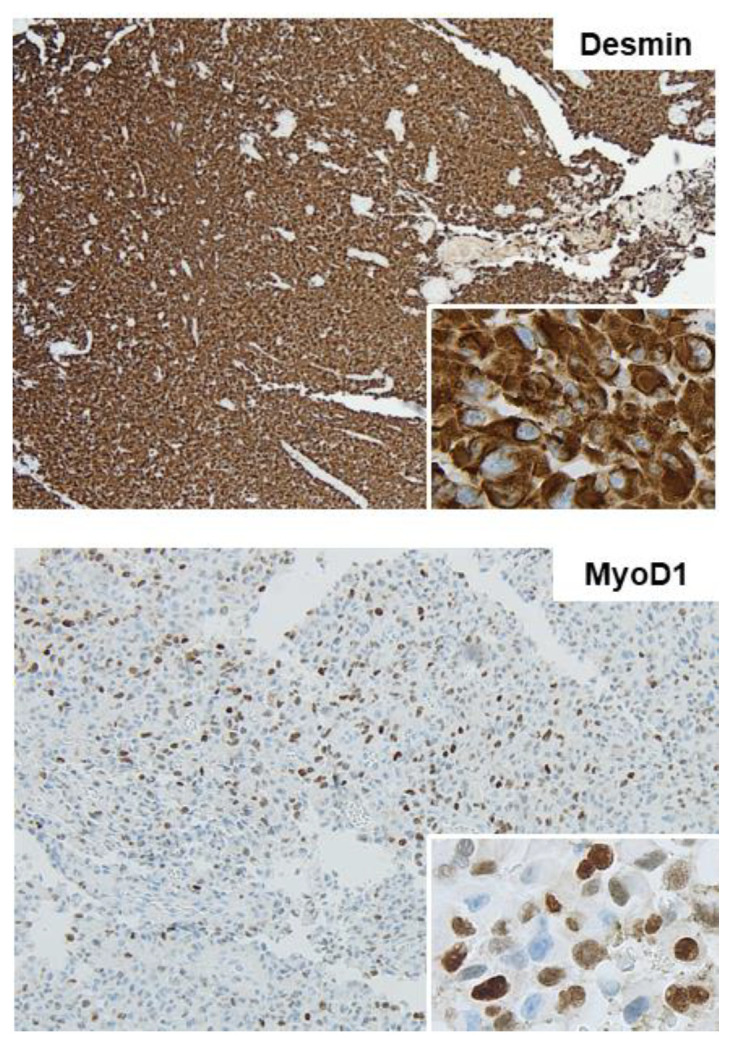
Skeletal muscle differentiation was proved with Desmin and MyoD1 staining.

Lymph nodes were tumor-free. Results of histological analysis showed an erosive lesion with a monomorphic cell population containing small cells with prominent nucleoli (Figure 1), and immunshistochemical stain showed negativity for cytokeratins (MN116). Only individual cells showed reactivity for MNF116-Stain, which excluded the diagnosis of undifferentiated or sarcomatoid carcinoma. Expression of INI-1 and melanoma markers (Sox-10 and S-100) was not detected. A positivity was confirmed for CD10, Vimentin, and Desmin. MyoD1 was detected, as well as a fluctuating signal for p53 (Figure 2 and Figure 3).

Molecular analysis revealed a negativity for Sox-10, and a weak positivity for CK8/18 by absence of p40. Chromosome 22q12 and absence of chromosome 16p11.2 were weakly detected. Being up to 90%, anti-tumor activity was considered high (Ki67).

RNA sequencing was evaluated by means of extraction and fragmentation of 28S rRNA transcripts. RNAseq libraries were prepared using Illumina TruSight RNA Fusion Panel (Illumina) and Next Generation Sequencing (MiniSeq, Illumina). Analysis of 1.033.545 reads showed a fusion of MYH9-SCD, which has not yet been described in the literature. Based on the morphological and immunohistochemical findings, the tumor was diagnosed as epithelioid RMS.

## 4. Discussion

Epithelioid RMS has been recently described in the literature as a subgroup of rhabdomyosarcoma tumors, which mainly affect adults and present an aggressive behavior. In contrary to another rhabdomyosarcoma subtypes (embryonal, alveolar, pleomorphic), epithelioid RMS commonly arises in the dermis without involvement of deep soft tissues. Since only 35 cases have been reported to date, it is considered a rare condition, which is not yet well characterized in the literature [1,2,3,4]. 

Due to its histological similarity with poorly differentiated carcinoma, it is suggested that epithelioid RMS tumors are diagnosed and treated as an epithelial tumor. Histologically, cells showing a sheet-like growth with prominent nucleoli and a high proliferation rate suggest carcinoma or melanoma cells [2,5]. The morphological appearance of the cells makes diagnosis difficult, and an accurate morphological and immunhistochemical analysis are required.

The myogenic lineage was confirmed based on the co-expression of Desmin and focal expression of Myogenin and MyoD1, which led to the diagnosis of epithelioid rhabdomyosarcoma. A fusion of MYH9 and SCD was detected. According to the authors’ knowledge, this variation has not been previously described in the literature, which hampers the interpretation of its significance [7,8]. 

Immunhistochemical analysis is the main component to differentiate epithelioid RMS from another rhabdomyosarcoma variants. Due to complex structural arrangements, molecular analysis remains inconsistent, and further investigations are required to understand its molecular signature [1]. An accurate diagnosis should consider histopathological features and their correlation with clinical conditions.

Differential diagnosis of epithelioid RMS includes other aggressive epithelioid tumors, such as poorly differentiated carcinoma and melanoma. Whereas Desmin is characteristic of soft tissue tumors, rhabdoid features can be present in other tumors, e.g., melanoma or myoepithelial carcinoma. Histopathologically, the absence of S-100 and SOX-10 differs between epithelioid RMS and melanoma [3,4,9]. Absence of endothelial markers (CD31 and ERG) in epithelioid RMS differentiates RMS from epitheloid angiosarcoma [3].

In accordance with the clinical features described in this report, epithelioid RMS tends to affect adult males, involving especially neck and head regions [2,4]. The tumor shows high proliferative activity, which makes its prognosis questionable, regardless of the surgical treatment provided. At the moment of diagnosis, patients present in a later stage of disease, and metastases to the liver, lung, and bone have been reported [2,3,10]. In this case report, lymph nodes were tumor-free and no metastases were diagnosed. Due to the lack of follow-up, no information can be provided about the current status of the patient. 

In summary, diagnosis of epithelioid RMS can be challenging due to the combination of epithelioid histological features with skeletal muscle differentiation. Thus, immunohistochemical and molecular findings are essential for the final diagnosis of epithelioid RMS, which includes co-expression of Desmin and markers of skeletal muscle differentiation, such as MyoD1.

## 5. Conclusions

Based on the morphological and immunohistochemical findings, the tumor was diagnosed as epithelioid RMS.

## Data Availability

Not applicable.
